# A Proof-of-Concept of Label-Free Biosensing System for Food Allergy Diagnostics in Biophotonic Sensing Cells: Performance Comparison with ImmunoCAP

**DOI:** 10.3390/s18082686

**Published:** 2018-08-15

**Authors:** Rocio L. Espinosa, María Fe Laguna, Fátima Fernández, Beatriz Santamaria, Francisco Javier Sanza, Maria Victoria Maigler, Juan J. Álvarez-Millán, Víctor Canalejas-Tejero, Miguel Holgado

**Affiliations:** 1 Center for Biomedical Technology, Optics, Photonics and Biophotonics Lab., Universidad Politécnica de Madrid, Campus Montegancedo, Pozuelo de Alarcón, 28223 Madrid, Spain; rocio.lopez@ctb.upm.es (R.L.E.); beatriz.santamaria@ctb.upm.es (B.S.); m.maigler@biod.es (M.V.M.); victor.canalejas@ctb.upm.es (V.C.-T.); m.holgado@upm.es (M.H.); 2 Department of Applied Physics and Materials Engineering, Escuela Técnica Superior de Ingenieros Industriales, Universidad Politécnica de Madrid, C/José Gutierrez Abascal 2, 28006 Madrid, Spain; 3 CQS Laboratory, Calle Marie Curie, 5, Rivas-Vaciamadrid, 28521 Madrid, Spain; ffernandez@cqssalud.com (F.F.); jamillan@cqslab.com (J.J.Á.-M.); 4 BioOptical Detection SL, Centro de Empresas, Campus Montegancedo, 28223 Madrid, Spain; fj.sanza@biod.es

**Keywords:** label-free biosensor, nitrocellulose, IgE, calibration curves, ImmunoCAP^®^, allergy diagnostic

## Abstract

Food allergy is a common disease worldwide with over 6% of the population (200–250 million people) suffering from any food allergy nowadays. The most dramatic increase seems to be happening in children and young people. Therefore, improvements in the diagnosis efficiency of these diseases are needed. Immunoglobulin type E (IgE) biomarker determination in human serum is a typical in vitro test for allergy identification. In this work, we used a novel biosensor based on label-free photonic transducers called BICELLs (Biophotonic Sensing Cells) for IgE detection. These BICELLs have a thin film of nitrocellulose over the sensing surface, they can be vertical optically interrogated, and are suitable for being integrated on a chip. The BICELLs sensing surface sizes used were 100 and 800 µm in diameter. We obtained calibration curves with IgE standards by immobilizating anti-IgE antibodies and identified with standard IgE calibrators in minute sample amounts (3 µL). The results, in similar assay format, were compared with commercially available ImmunoCAP^®^. The versatility of the interferometric nitrocellulose-based sensing surface was demonstrated since the limit of detections for BICELLs and ImmunoCAP^®^ were 0.7 and 0.35 kU/L, respectively.

## 1. Introduction

Food allergy is an immune-based disease that has become a serious health concern worldwide. The prevalence and severity of food allergy has dramatically increased in both developed and developing countries in the last 15 years [1,2,3]. Thus, around 200–250 million people suffer from any kind of food allergy [4], affecting more than 17 million people in Europe alone. Three million out of the 17 million are allergic patients younger than 25 years old. The rise is of special concern for children and young people due to the increased number of life-threatening allergic reactions [5]. For this, it is important to improve the diagnosis of these diseases to implement treatments and easy monitoring as soon as possible.

Current diagnostic methods for food allergies might be classified in three levels [1]. The first-level methods combine the medical history with in vivo tests, such as a skin prick test (SPT), but without a definitive diagnostic of food allergy [6] and with some market limitations for allergen extracts used in SPT [7]. The second-level comprises in vitro tests, specifically blood analysis, normally based on detection of immunoglobulins type E (IgE) in human serum, measuring their specific response to allergens either from food extracts (in vitro assays IgE) or from isolated molecular allergens, such as in vitro component resolved diagnosis (CRD). In both in vitro methods, classic immunochemical techniques are used. The third-level method is the oral provocation test (OPT). OPT is the most effective method to diagnose a food allergy; however, OPT is required to be performed by personnel trained in resuscitation procedures and in a clinical setting with the adequate equipment and drugs in case of an emergency [6]. From these three, in vitro CRD tests seem to be the most promising method for accomplishing a safe and accurate food allergy diagnosis [8,9,10].

Commercial in vitro tests use immunochemical techniques based on immobilization of the allergen on solid phase or over different surfaces, such as microplates, microarrays, capsules, and membranes. The sample (human serum) is incubated over the surface, waiting for the IgE binding of those components to which the patient is sensitized. After that, the binding is visualized through the optical signal that is developed by means of a labeled secondary antibody with enzymes, fluorophores, or gold nanoparticles. A relevant limitation of these techniques is the difficulty of standardization of the allergens used in diagnosis when food extracts are used [8,11,12]. Another drawback is the lack of parallelism between calibration and sample curves due to the unavailability of specific IgEs for calibration. Although these techniques are commonly used in daily practice, their different working format makes the quantification inaccurate and the comparison between systems is poor [13,14,15]. Finally, the need of several steps, labeled reagents, and specific and non-portable laboratory equipment are other relevant disadvantages for these techniques due to their high costs.

The point-of-care (PoC) devices are an innovative cost-effective alternative able to solve some of the aforementioned limitations [16]. However, PoC solutions in the market can only provide a qualitative measurement of the presence of a limited number of specific IgE antibodies. Besides the inability of providing personalized component-resolved sensitization, commercial PoCs do not solve problems behind the use of crude extract, labeled reagents, and heterologous interpolation of a sample signal.

Considering the abovementioned reasons, we proposed the use of a cost-effective label-free detection system as an outstanding alternative to in vitro CRD tests. Our diagnostic system was based on disposable biochips with the capability of implementing hundreds of our patented biosensing sites (Biophotonic Sensing Cells (BICELLs) [17]) on them. The read-out platform works in a label-free format by reading the interferometric signal of each BICELLs and measuring the binding events that take place on them. This novel method for interrogating the sensing sites was published [18] and previously patented at the European level [19]. The whole system has already been proven in other bioapplications such as dry-eye, Bovine Serum Albumin (BSA)/anti-Bovine Serum Albumin (aBSA), and anti-gestrinone antibodies [20,21,22,23]. In this paper we followed the calibration strategy for clinical IgE detection assays [11], and assessed the workability of BICELLs in allergy diagnosis with a minute amount of sample (3 µL for BICELL vs 40 µL required for ImmunoCAP). Lastly, in this paper we report some preliminary results with two BICELLs’ sizes for detecting IgE molecules from standard ImmunoCAP calibrators over their surfaces functionalized with anti-IgE. Control results proved the selectivity of the sensing surface and the lack of binding gaps. A detailed and rigorous study about measuring IgE antibody levels against molecular allergens, the BICELLs’ size, volume, sensitivity, and readout technology involved will be published elsewhere.

In this study, as proof of concept for allergy biomarker detection, we compared our IgE calibration curves with the in vitro “gold standard” for the analysis of specific immunoglobulins in human serum samples, the ImmunoCAP^®^. We obtained calibration curves for two different sizes of BICELLs 100 and 800 µm in diameter. We biofunctionalized BICELLs with anti-IgE antibodies and measured increasing levels of IgE molecules in the recognition step. The minute amount of sample required was 3 µL for BICELL. We accomplished the recognition response curve by optical readout with the already tested methodology of a novel label-free point-of-care (PoC) reader [24], and compared both curves. The comparison between calibration curves allowed us to evaluate our system with promising results due to the limit of detection (LOD) obtained.

## 2. Materials and Methods

### 2.1. BICELLs Fabrication and Materials

We used integrated biochips that were made up of three interferometric Biophotonic Sensing Cells (BICELLs). BICELLs are based on a Fabry-Perot interferometer (FPI), and its optical label-free biosensing capability is enhanced by adding a thin film of nitrocellulose over the two interferometric layers of SiO_2_ and SU-8 photoresist (MicroChem Corp., Westborough, MA, USA) [25,26]. We have previously reported the novelty of fabricating at wafer level and the final step of spin coating to deposit nitrocellulose (Sigma-Aldrich, St. Louis, MO, USA) [27].

We used a 4″, p-doped, <100> Si wafer (SIEGERT WAFER GmbH, Aachen, Germany) with a 140 nm thermal oxide on top as substrate for fabricating our BICELL interferometer. A 420 nm thin layer of SU8 2000.5 resist was spun onto the substrate by a conventional spin coating technique. Prior to SU8 exposure using a standard optical mask aligner, a 30′@115 °C soft bake in a hot plate was performed. We used an exposure time of 80’ to pattern both 800 and 100 µm BICELLs, followed by a crosslinking bake of 5’@115 °C and development. To create the last layer of the biosensor, the nitrocellulose was spun over the SU8 cells and exposed by deep ultraviolet (DUV) with a quartz mask, inversely copied from the mask used in the first lithography step, and immersed in AR-600-55 (ALLRESIST GmbH, Straugsberg, Germany; 1:4 in isopropanol) developer, rinsed distilled-water and dried with N2.

**Figure 1:**
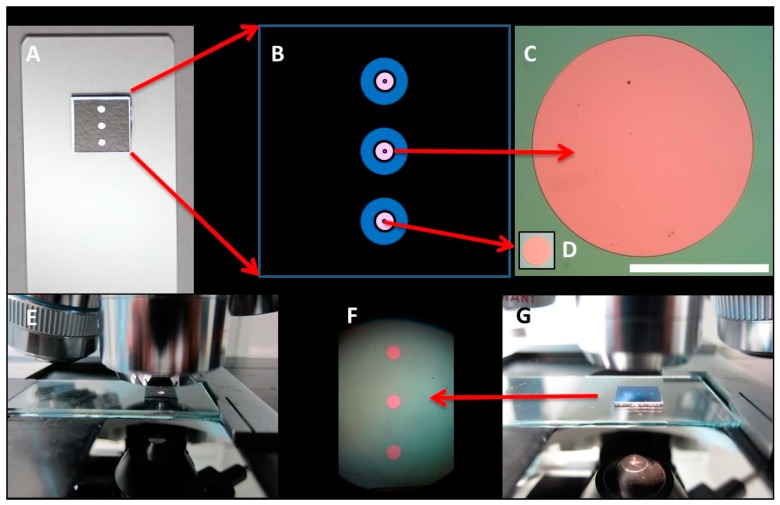
800 and 100 µm BICELLs. (**A**) 100 µm BICELLs integrated in biochips with vinyl sticker; (**B**) Schematic design of 100 and 800 µm BICELLs; (**C**,**D**) Detail of 800 and 100 µm BICELL (100 µm image is overlaid on the 800 µm image. Bar scale is equal to 500 µm); (**E**) Morphological characterization of 100 µm BICELLs; (**F**) Three 800 µm BICELLs integrated in a biochip; (**G**) Morphological characterization of 800 µm BICELLs (morphological characterization of BICELLs was carried out using an optical microscope Leica Leitz DMRX).

Once the integrated biochips were fabricated, vinyl stickers and glass substrates accomplished the final kit packaging, as shown in Figure 1. The hydrophobic behavior of the vinyl and the three wells fluidic pattern allowed the dropping and restraining of liquid samples over BICELLs.

### 2.2. Optical Label-Free Characterization of BICELLs

**Figure 2:**
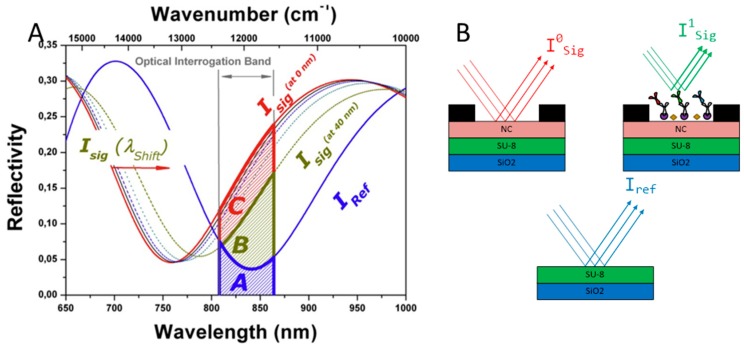
Optical label-free sensing mechanism with BICELLs. (**A**) Theoretical calculation of the reflectivity as a function of the wavenumber and wavelength for the reference interferometer and for the signal interferometer. (**B**) Optical scheme of BICELLs without biomolecules (top-left), after the recognition step (top-right), and optical power of the reference interferometer (bottom). Image (**A**) comes from Reference [18].

In order to accomplish our methodology, we obtained the Increased Relative Optical Power (IROP) signal in both type of BICELLs with the optical interrogation PoC reader already mentioned. The theoretical sensing principle behind this system has been previously explained in detail [28,29]. Briefly, the theoretical calculation of the reflectivity is a function of the wavenumber and wavelength for the reference interferometer and for the signal interferometer. The areas (capital letters A, B, and C in Figure 2A) are defined by the optical interrogation band and the interferometric profile of the FPI used. These areas are proportional to the optical power in different situations. Figure 2B top shows the optical scheme of BICELLs when the biofilm thickness is 0 nm (I^0^_sig_ and area C from Figure 2A) and when biomolecules are immobilized on the sensing surface with a biofilm thickness bigger than 0 nm (I^1^_sig_ and area B). Figure 2B bottom shows the optical power of the reference interferometer (I_ref_ and area A from Figure 2A). As a conclusion, an increase on the biofilm thickness is directly related to a FPI spectrometry profiles approach and therefore, to a lower level of IROP signal.

We used the label-free PoC reader and the readout optical method to optically interrogate BICELLs. The optical response of the PoC is the ΔIROP (%) value obtained as the quotient of the optical power of two interferometers: the signal and the reference interferometers [18,28] (Equation (1)).

(1) $$\Delta_{\mathrm{IROP}}={\mathrm{IROP}}_{1}-{\mathrm{IROP}}_{0}=\left[\left(\frac{I_{\mathrm{Sig}}^{1}}{I_{\mathrm{Ref}}}\right)-1\right]\times 100-\left[\left(\frac{I_{\mathrm{Sig}}^{0}}{I_{\mathrm{Ref}}}\right)-1\right]\times 100$$

Equation (1): ΔIROP equation. ΔIROP is the difference between the IROP_0_ at 0 nm of biofilm thickness and the IROP_1_ at a given biofilm thickness. Equation (1) comes from Reference [18].

### 2.3. ImmunoCAP^®^ Protocol for Obtaining of Calibration Curves Anti-IgE/IgE

We performed the reference IgE calibration curves with ImmunoCAP^®^ equipment (model UniCAP 100 E), where fluorescence immunoassays were developed. Briefly, we incubated increasing concentration of IgE calibrators (40 µL, 37 °C) over cellulose CAPs functionalized with an anti-IgE monoclonal antibody (time needed to functionalized cellulose CAPs is not specified for the provider). For the next steps, we added secondary labeled antibody-β-galactosidase anti-IgE-(50 µL, 37 °C), and after, the substrate solution (50 µL, 37 °C) for fluorescence development. Finally, we added a stop solution (600 µL, 37 °C), and the signal assay was measured. The total time fir the processing assay was 2.5 h. All the reagents and solutions were purchased from Thermo Fisher Scientific/Phadia, Uppsala, Sweden. The measurements were performed in duplicate (*n* = 2 caps).

The UNICAP built a curve for the low IgE concentration range (0.35–100 KU/L) and another for higher IgE ranges (2–5000 KU/L). The IgE calibrators were human IgE biomolecules at increasing concentrations in a pH 7 buffer.

### 2.4. Point of Care Protocol for Obtaining of Calibration Curves Anti-IgE/IgE

We developed an oriented immunoassay model based on the pair anti-IgE/IgE to evaluate the standard calibrators in our system and compare it with ImmunoCAP^®^. We biofunctionalized BICELLs with an anti-human IgE, which was a mouse monoclonal antibody (Abcam, Cambridge, UK). We used ProteinA (Sigma-Aldrich, St. Louis, MO, USA) as a linker between nitrocellulose and anti-IgE to help anti-IgE to be correctly oriented. Bindings were accomplished using strong electrostatic forces (nitrocellulose-ProteinA) and by using affinity in the pair ProteinA-anti-IgE through a long time of incubation. We used Bovine Serum Albumin (BSA) (Sigma-Aldrich, St. Louis, MO, USA) as a blocking agent to prevent nonspecific binding on the remaining binding surface. Finally, we measured the recognition response in increasing levels of human IgEs molecules provided by ImmunoCAP^®^ calibrators (ThermoFisher Scientific, Phadia AB, Uppsala, Sweden) and compared both recognition responses (i.e., ImmunoCAP^®^ and our system responses).

**Figure 3:**
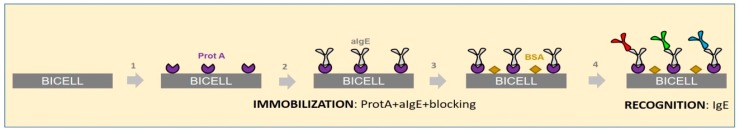
Anti-IgE/IgE protocol. 1–3: Immobilization step (1. Protein A; 2. anti-Immunoglobulin E (aIgE); 3. Blocking with Bovine Serum Albumin (BSA)). 4. Recognition step with Immunoglobulin E (IgE).

The immobilization and recognition steps for both sensing surfaces (800 and 100 µm) are shown in Figure 3. Before biofunctionalizing the kits, we activated the nitrocellulose surface by washing BICELLs with 20 mL of micro-filtered distilled Mili-Q water and blowing them with clean and particle-less air. We also established a cleaning protocol with two different steps. First, kits were manually washed with micro-filtered Mili-Q water or with phosphate buffered saline PBS-Tween (1:100 Sigma-Aldrich, St. Louis, MO, USA). We used polyethersulfone PES 0.45 µm filters and syringes, and we varied the amount of water/PBS-T depending on the analyte incubated on the surface of the kit. Second, kits were dried with dust free clean air for a few seconds, just to eliminate humidity from the surface.

We established the immobilization stage for the oriented antibody by incubating ProteinA (50 µg/mL prepared in distilled MiliQ-water; 3 µL/cell for 30 min at 38 °C in a humid environment). Kits were then incubated with anti-IgE (50 µg/mL in PBS 1:100; 3 µL/cell for 14 h at 36 °C in a humid environment), and blocked with BSA (3% in PBS 1:100, 3 µL/cell, 15 min at 38 °C). The cleaning protocol applied was 30 mL of H_2_O for ProteinA, 20 mL of PBS-T and 10 mL of H_2_O for anti-IgE, and 60 mL of PBS-T and 30 mL of H_2_O for BSA.

We developed a recognition protocol for accumulative immunoassays by incubating different concentrations of ImmunoCAP^®^ IgE calibrators. First, we performed accumulative immunoassays with increasing concentrations of calibrators in the range of 2–5000 kU/L (specifically 2, 10, 50, 200, 1000, and 5000 kU/L) for 800 µm BICELLs, and second, we used calibrators in the range of 0.7–1000 kU/L (specifically 0.7, 2, 3.5, 17.5, 50, 100, 200, and 1000 kU/L) for 100 µm BICELLs. We incubated 3 µL/cell for 20 min at 36 °C in a humid environment, and then applied the cleaning protocol established (30 mL of H_2_O for low concentrations [0.7–50 kU/L]; 60 mL of H_2_O for high concentrations [100–5000 kU/L]). The measurements were performed in 13 biochips (*n* = 39 BICELLs) with 6 and 7 biochips for 800 and 100 µm BICELLs, respectively. The immobilization stage took 15 h 30 min (incubation and data processing) and each step of the accumulative assay took just 40 min (20 min and 20 min of incubation and processing). The total time of the assays was 19 h 30 min and 20 h 50 min for 2–5000 kU/L and 0.7–1000 kU/L, respectively.

We implemented two control experiments: a negative control to check the non-affinity between BSA and IgE, and a negative control to study the selectivity of the sensing system, for 800 and 100 μm BICELLs, respectively. We accomplished BSA control by immobilizing BSA (3% in PBS 1:100, 3 µL/cell, 15 min at 38 °C) on the surface and identified with a 10 kU/L IgE calibrator (3 µL/cell for 20 min at 36 °C in a humid environment). We also included a negative control for surface selectivity by immobilizing aIgE in the same conditions as for non-control experiments at the immobilization stage, and performing a recognition step with protein MMP9 (Sino biological Inc., Beijing, China) in a concentration of 75 ng/mL (equivalent to almost 30 kU/L [30]).

## 3. Results and Discussion

Despite the drawbacks of CRD commercial devices, they are commonly used in the daily practice for food allergy diagnosis. In line with the recommendations made by the American Academy of Allergy, Asthma and Immunology (AAAAI) for further studies [6], and the need of consolidating immunoassays with other assay technologies [16], we developed a proof-of-concept assay comparing our calibration curve with the calibration curve obtained by ImmunoCAP^®^, with promising results of LOD of 0.7 kU/L (very close to the ImmunoCAP^®^ LOD of 0.35 kU/L and to other LODs reported in [31,32]).

### 3.1. Calibration Curves Obtained by Optical Label-Free Technique

BICELLs are interferometric biotransducers whose optical response changes when some biological reaction occurs on their surfaces. In the absence of a label, when a biological component is immobilized or recognized on the sensing surface of a BICELL, the biological reaction is directly detected by reading a change in the refractive index of the surface [28]. The optical label-free detection methodology used can be applied for detection of antigens or antibodies. Moreover, fewer steps are required, since labels as enzymes or fluorophores are not needed in the proposed biosensor, making our technology more advantageous than classic immunochemical techniques (ELISAs, microarrays, etc.). Apart from the photonic transducer properties, the biosensing system depends on the read-out optical method employed.

We obtained the calibration curve by measuring the ΔIROP (%) response for different concentrations of IgE calibrators. In order to analyze analyte-receptor recognition reactions, we evaluated the ΔIROP (%) in each step of the immunoassay. Taking into account that the interferometric signal came from the biomolecules adsorbed on the surface of BICELLs, there was a great interest in avoiding nonspecific binding onto them. Indeed, we accomplished a label-free recognition step due to the effort made to fully cover BICELL surfaces with anti-IgE and BSA molecules at the biofunctionalization stage. Experimental results illustrated that the interferometric peak decreased as the biomolecules concentration increased. In this work, we proved the biosensing surface with BICELLs of 800 µm, and then we reduced the size to 100 µm to enhance their sensibility. Such a size reduction allowed us to have a recognition step in lower concentrations of IgEs. It is important to remark that the ΔIROP (%) signal in calibration curves represents the difference between ΔIROP (%) at the specific concentration in the recognition stage and the ΔIROP (%) at the blocking step; hence, it is an absolute value of ΔIROP (%).

**Figure 4:**
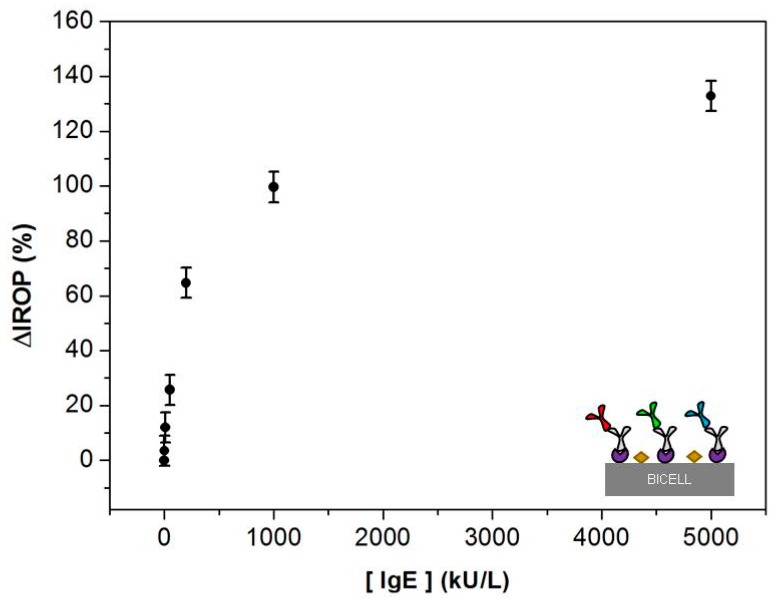
Sensing recognition curve with 800 µm BICELLs. Calibration curve with Increased Relative Optical Power (∆IROP (%)) vs concentration of IgE calibrators in the range (2–5000 kU/L). *n* = 18 BICELLs.

For 800 µm BICELLs, the sensing recognition curve (Figure 4) shows ΔIROP (%) as a function of the concentration of IgE calibrators in the range 2–5000 kU/L. We obtained a ΔIROP of 11.95% at 10 kU/L (equivalent to 24 ng/mL [30]). This result suggests that 800 µm BICELLs biofunctionalized with an anti-IgE antibody allowed for binding of IgE molecules in concentrations as low as 24 ng/mL. Likewise, four concentration points were included in the increasing linear region of the curve, indicating that IgE molecules were binding with available anti-IgE bioreceptors. Finally, saturation seemed to occur near a 5000 kU/L concentration with a ΔIROP of 132.8%.

**Figure 5:**
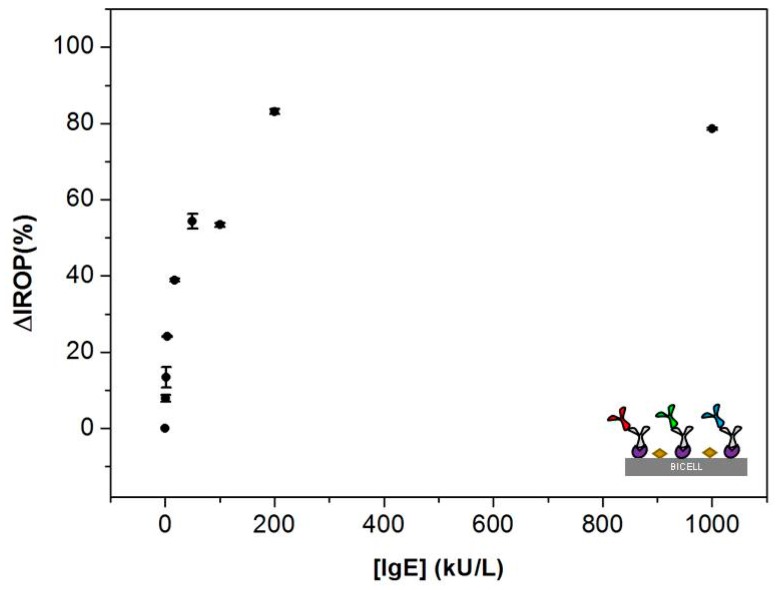
Sensing recognition curve with 100 µm BICELLs. Calibration curve with Increased Relative Optical Power (∆IROP (%)) vs concentration of IgE calibrators in the range (0.7–1000 kU/L). *n* = 21 BICELLs.

For 100 μm BICELLs, the sensing recognition curve shown in Figure 5 illustrates the ΔIROP (%) signal as a function of the concentration of IgE (range 0.7–1000 kU/L) incubated in each step. The experimental analytical linear range found for IgE recognition, in which we were able to detect quantitative response, began at 0.7 kU/L with a ΔIROP (%) of 7.86% and a flat stage was reached at around 200 kU/L (83.01% ΔIROP). The ΔIROP (%) signal remained almost unchanged for 200 and 1000 kU/L, indicating that the anti-IgE bioreceptors were almost fully saturated. These preliminary results indicated that a reduction in BICELLs size enhanced the sensitivity of the system as expected. The reason is because a lower concentration of target biomolecules covered a higher percentage of the transducer sensing area. In other words, this smaller sensing area permitted the saturation of the sensing surface for lower concentration of biomolecules.

**Figure 6:**
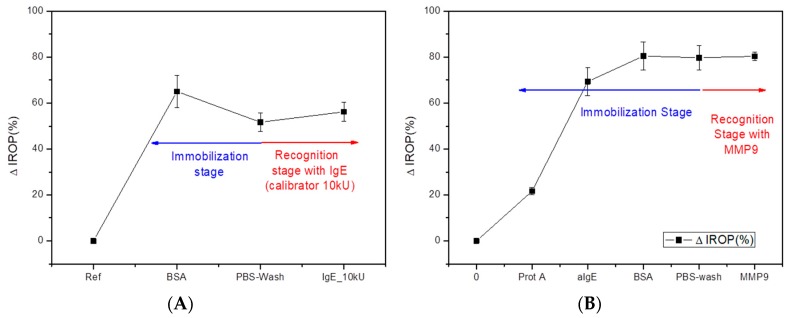
Results for negative control experiments. (**A**) ∆IROP (%) in a control experiment with BSA. ∆IROP (%) for BSA, PBS-wash step, and recognition step with IgE [10 kU/L]; (**B**) Complete experimental curve for negative control experiment with MMP9 at recognition step. Y axis represents absolute ∆IROP (%) signals.

Results for control experiments are shown in Figure 6. Figure 6A is for the negative control with BSA. In this chart there is a decrease in the ΔIROP (%) signal at the end of the immobilization stage and a small rise after incubating IgE calibrators. The slight decay in the absolute ΔIROP (%) signal after washing with PBS is an indication of the BSA-nitrocellulose binding strength. Likewise, the minor increase at the recognition stage is an experimental demonstration of both the non-affinity between BSA and IgE, and the total coverage of the sensing surface with BSA. Thus, the selectivity of the target biomolecules is proved if the blocking agent works correctly.

Figure 6B represents the complete experimental curve (immobilization and recognition stages) with its ΔIROP (%) signals for the negative control with MMP9. In this chart we illustrate the increase in the absolute ΔIROP (%) signal as the biofilm thickness grew in each step. The ΔIROP (%) reached an 80% IROP (%) in the blocking step and remained almost unchanged after washing with PBS. The stabilization of the ΔIROP (%) signal even after incubating MMP9 demonstrated the selectivity of the sensing system. Likewise, the strength of the binding ProteinA-aIgE was proved. A dissociation of bindings would be seen as a fluctuation in the ΔIROP (%) signal, but the stability remained after the recognition step.

### 3.2. Comparison of the ImmunoCAP^®^ Versus the Optical Label-Free Technique Calibration Curves

**Figure 7:**
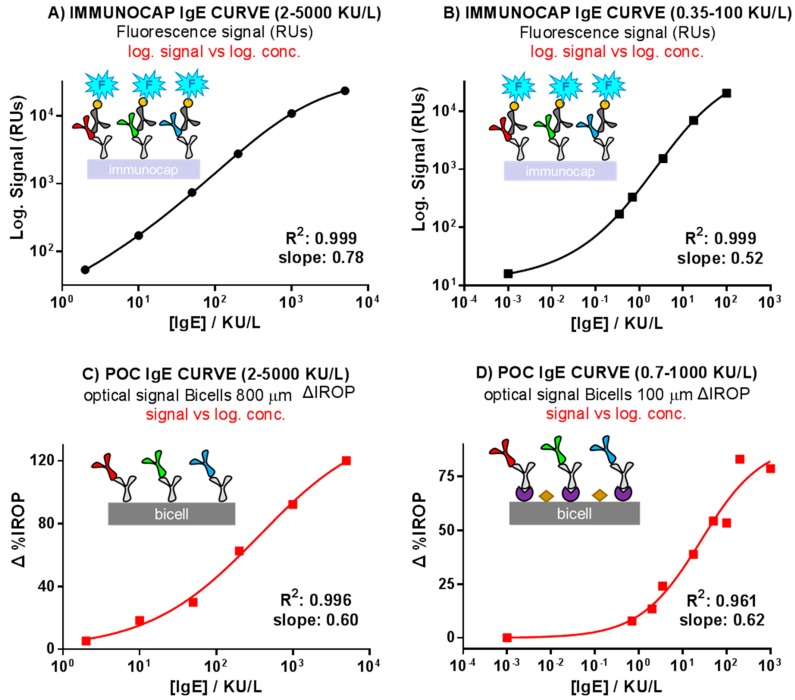
ImmunoCAP^®^ and biosensor calibration curves. (**A**) ImmunoCAP^®^ curve, range 2–5000 kU/L; (**B**) ImmunoCAP^®^ curve, range 0.35–100 kU/L; (**C**) Biochip curve, range 2–5000 kU/L (point-of-care (PoC) reader platform signal); (**D**) Biochip curve, range 0.7–1000 kU/L (PoC reader platform signal).

ImmunoCAP^®^ is considered the gold standard diagnostic technique for food allergies [33], however, its quantification methodology is performed in a different working format for calibrators than for samples. Hence, in order to achieve the validation of the system proposed for us and to compare both recognition responses, we accomplished the comparison of our results with the ImmunoCAP^®^ curves working in a similar assay format. The comparative results are shown in the curves of Figure 7. Sigmoidal adjustments were applied in both cases, representing the X as log of the concentration. Two IgE concentration ranges, i.e., 2–5000 kU/L (graph A) and 0.35–100 kU/L (graph B), are shown for ImmunoCAP^®^. In the case of the biochip, the response of the PoC reader with 800 µm BICELLs (concentration range: 2–5000 kU/L) and with 100 µm BICELLs (concentration range: 0.7–1000 kU/L) are shown in graph C and D, respectively.

The response of the biochips was sensitive and with good curve correlations (R^2^ > 0.95). The LOD reported (0.7 kU/L, equivalent to 1.68 ng/mL [30]) is the low concentration of IgE detectable in 100 µm BICELLs. Thus, the R^2^ and the dynamic range were calculated for the ImmunoCAP and BICELLs’ more sensitive curves (i.e., Figure 5, Curves B and D). Dynamic ranges for a 20–80% normalized curve were 0.09–11.71 kU/L and 2.38–133.12 kU/L for Curves B and D, respectively). It should be noted that, for the moment, this experimental LOD level is close to the LOD of ImmunoCAP^®^ (0.35 kU/L) and to other biosensors LODs reported in References [31,32] (specifically 0.6 ng/mL and 2.5 ng/mL of IgE). Likewise, the detection with the BICELLs-based biochip was much simpler, since two fewer steps were necessary than when using ImmunoCAP^®^ (specifically, the secondary labelled antibody and fluorescent substrate steps).

## 4. Conclusions

In this work, we performed a biofunctionalization strategy of BICELLs in order to obtain a calibration curve comparable to the ImmunoCAP^®^ one. Indeed, we implemented a common immunoassay protocol for both sizes of BICELLs in an attempt to establish a standard biochemical procedure. To the best of our knowledge, we have demonstrated, for the first time, highly sensitive molecular recognition for BICELLs consisting in a layer of SU-8 over SiO_2_ wafers with a thin film of nitrocellulose, tested in real conditions with anti-IgE for detecting increasing concentrations of IgE molecules. The versatility of the interferometric nitrocellulose-based sensing surface was demonstrated since the LOD for BICELLs was as low as 0.7 kU/L, being close to the ImmunoCAP^®^ LOD (0.35 kU/L). The following step will be to demonstrate the applicability of the proposed system for detection of specific IgEs in human serum samples by using BICELLs biofunctionalized with molecular allergens.
